# Dietary inclusion of royal jelly modulates gene expression and activity of oxidative stress enzymes in zebrafish

**DOI:** 10.1080/14756366.2021.1900167

**Published:** 2021-03-23

**Authors:** Ercüment Aksakal, Deniz Ekinci, Claudiu T. Supuran

**Affiliations:** a Faculty of Agriculture, Department of Agricultural Biotechnology, Division of Animal Biotechnology, Akdeniz University, Antalya, Turkey; b Faculty of Agriculture, Department of Agricultural Biotechnology, Ondokuz Mayıs University, Samsun, Turkey; c Neurofarba Department, University of Florence, Polo Scientifico, Sesto Fiorentino (Firenze), Italy

**Keywords:** Royal jelly, antioxidant enzymes, zebrafish, nutrition, inhibition, gene expression

## Abstract

Here we investigated the effects of different levels of royal jelly in zebrafish (*Danio rerio*) diets [0.0% (D1); 0.1% (D2); 0.4% (D3); 1.6% (D4) vs 6.4% (D5)] on the activity and expression profiles of superoxide dismutase, catalase, glutathione reductase, glutathione peroxidase and glutathione S-transferase. Muscle, liver and kidney tissue samples were obtained from fish fed during 8 weeks. In these tissues, enzyme activity was determined by means of spectrophotometer and gene expression by quantitative real-time PCR. mRNA levels of the enzymes were elevated in almost all diet groups compared to the control (D1). It was determined that enzyme activities were also increased in general by supplementation of royal jelly although some decreases were also observed. However, the significant correlation between gene expression and enzyme activity was not observed in all tissues. It was concluded that main regulation occurs with post-translational modifications although effects at transcriptomic level demonstrated a snap variation.

## 1. Introduction

Royal jelly, which is the richest nutrient among bee products, was first reported in 1623 to be produced only for the queen bee. In the past, the term "Gelatine Reale", meaning royal-specific, was used for this substance, but today this term is used as "Royal Jelly (RJ)"^1^. The royal jelly is secreted by the intracranial glands and pharynx glands of young worker bees to feed young larvae and adult queen bees.

The sugar content in royal jelly consists of a significant amount of glucose and fructose and a low level of sucrose. The glucose level in royal jelly is of great importance for the completion of muscle development in the larvae. The higher sugar level in royal jelly used for feeding young queen bee larvae enables queen bees to acquire stronger physical properties than other bee members. The carbohydrates found in royal jelly have a stimulating effect for nutritious bees and encourage more royal jelly production.

The quality of royal jelly is determined according to the 10-HDA ratio and this ratio is required to be between 1.4 and 1.8% in a qualified royal jelly. This ratio varies according to the vegetation of the region where the royal jelly is located and the applications in royal jelly harvesting^2^^,^^3^. The royal jelly contains mostly K, Na, Cu, Fe, Mn and Zn trace elements. Unlike honey, the mineral substance composition in the royal jelly is not affected much by the geographical origin and different plant variety^4^.

The main ingredients of royal jelly are water, protein, carbohydrate, lipid and mineral salts. Although the composition of royal jelly varies according to geographical and botanical conditions, it generally contains an average of 60–70% moisture, 12–15% crude protein, 10–16% sugar, 3–6% lipid and low molecular weight compounds (vitamins, salts and free amino acids) and water-soluble protein fractions. Royal jelly is used for various purposes in traditional medicine, apitherapy, cosmetic and pharmaceutical industries due to its strong biological activities. It has been reported that the antioxidative properties of the royal jelly have positive effects on human health against a number of diseases that may occur due to free radicals, especially cancer, coronary and inflammatory diseases, neurological degeneration and aging^5^^,^^6^.

The unique feature of royal jelly is due to the C8-C12 hydroxy group and dicarboxylic fatty acids^7^^,^^8^. It is known that 10-hydroxy-2-decanoic acid (10-HDA), the essential unsaturated fatty acid of royal jelly, has various pharmacological effects such as antibiotic^7^, antitumoral^9^, antioxidative^5^^,^^10^ and hypoglycaemic activities^11^. 10-HDA is used to distinguish and determine the freshness, quality and specificity of royal jelly. 9-hydroxy-2E-decanoic acid (9-HDA) in royal jelly can be considered as a metabolite of 9-oxo-2E-decenoic acid (9-ODA) produced by worker bees^12^. This compound is a well-known semi-chemical that has important functions such as the recognition of the queen bee and the prevention of ovarian development of worker bees for the maintenance of the class system in honeybee colonies.

Zebrafish (*Danio rerio*) lives mainly in Southeast Asia, the Ganges Region of East India, Pakistan, Bangladesh, Nepal and Myanmar. It exists more where water moves slowly (pools, ponds, lakes, canals and rice paddies). Zebrafish has been frequently used as a model organism in biological experiments. Advantages of zebrafish as a model organism are as follows; They are cheap, have sufficient resource accumulation, their biological stages are well known, they reproduce by external fertilisation and give many eggs under optimum conditions, they have easy maintenance under appropriate conditions, easy monitoring and manipulation of embryonic development, easy culture, they are also suitable for experimental manipulations in addition to having high homology with the human genome, and it is easy and convenient to study for genetic analysis^13^.

Aerobic tissues steadily produce reactive oxygen species (ROS) under physiological conditions, which are by products of oxidative metabolism^14^^,^^15^. ROS generation may be useful in some specific cell types in order to fight a pathogen. Nevertheless, generally, high levels of ROS in a cell lead to several injuries like DNA damage, inactivation of enzymes, structural protein degradation and polyunsaturated fatty acid (PUFA) peroxidation that cause alteration of development and pathologies^16^. Due to having high amounts of *n*-3 highly unsaturated fatty acids (HUFA), fish are susceptible to both lipid peroxidation and tissue damage based upon lipid peroxidation^17^. Overcome of prooxidants against antioxidant defences is the cause of oxidative stress in cells. Diet composition can worsen this unbalance. Fish feeds, especially the ones for larvae include high amounts of PUFAs, which are fatty acids having tendency to create oxidative damage during diet preparation and storage. Since the products obtained from lipid peroxidation are absorbed and transported to tissues, oxidative stress may be induced^18^. All aerobic organisms have two different systems for antioxidant defense to reduce ROS levels and avoid lipid peroxidation. One of them corresponds to enzymes which involve superoxide dismutase (SOD), catalase (CAT), glutathione reductase (GR) glutathione peroxidase (GPx) and glutathione S-transferase (GST). SOD is a metalloenzyme catalysing the dismutation of the superoxide anion (O_2_^−^) into oxygen and hydrogen peroxide (H_2_O_2_)^14^. Following this, CAT enzyme reduces H_2_O_2_ to water in the peroxisomes. Same reaction is catalysed by GPx as CAT in the cytosol. Additionally, GPx converts lipid hydroperoxides into a more stable form, which are lipid hydroxides. GR is responsible for conversion of oxidised glutathione (GSSG) to reduced form (GSH). In the reduced form, glutathione plays key roles in the cellular control of reactive oxygen species. GST is responsible for catalysing the conjugation of GSH to several xenobiotic substrates for the purpose of detoxification.

In order to investigate the mechanistic background of fish nutrition, it is necessary to have a full understanding of biochemical mechanisms that enable effective enzymatic catalysis under several nutritional conditions. In the literature, there is a lack of data on the biochemical outcomes of royal jelly feeding and no data has been received on its effects on zebrafish antioxidant system. Therefore, this study aimed to measure the effects of dietary royal jelly, which might be a novel source in animal feeding, on the activity and expression levels of antioxidant enzymes in muscle, kidney and liver tissues of zebrafish.

## 2. Materials and methods

### 2.1. Chemicals

All chemicals were purchased from Sigma-Aldrich, Munich, Germany or Merck, Darmstadt, Germany with analytical grade.

### 2.2. Fish, maintenance and ethical approval

**Figure 1.**
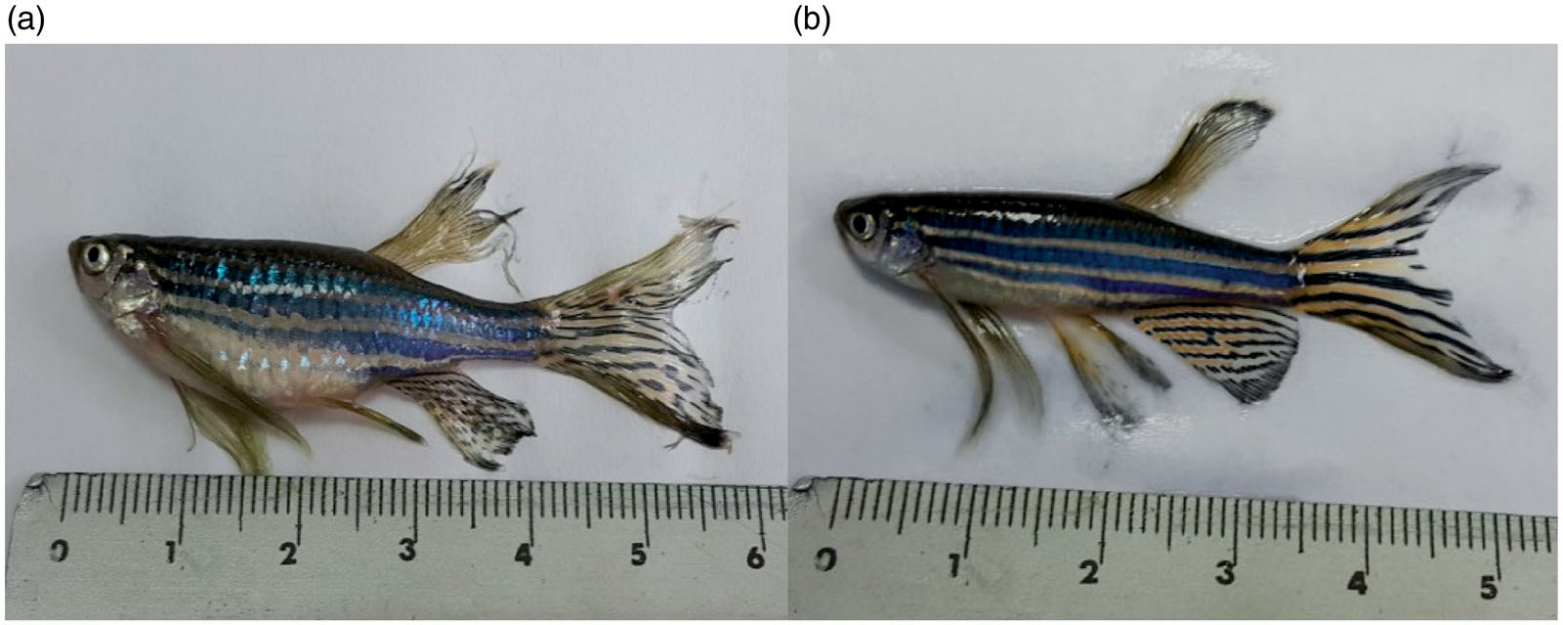
Female (a) and male (b) zebrafish (*Danio rerio*).

The experiment was conducted at the model organism unit and biotechnology laboratories at Agricultural Biotechnology Department of Ataturk University. A total of 250 (1♂:1♀) zebrafish (2-month-old and 0.35 ± 0.15 g) were used (Figure 1). The ethical approval for this study was obtained from the Atatürk University Ethics Comite with the approval number 36643897–82/65.

#### 2.2.1. Water material

Mains water in the model organism unit was used as the water material in the study. Before the water was supplied to the system, it was passed through eight different filters and aerated for 2 days in the settling tanks. The water parameters are given in Table 1.

**Table 1.** Chemical properties of water used in research

Parameter	Value
O_2_	9 ± 0,5 mg/lt ppm
pH	7.30
Saturation	%70–80
Temperature	26 ± 1.0 °C

#### 2.2.2. Model organism unit

Experimental works were carried out in a model organism unit, which is a Recirculating Aquaculture Systems (ARS-1500) (Akuamaks Company, Ankara, TURKEY). The advantages of the system are as follows: the temperature value required for the fish material is kept constant, the water is constantly circulated and the desired optimum conditions can be achieved.

#### 2.2.3. Diet material

In the study, five different gelatine and casein-based diets were prepared containing royal jelly in the ratios of D1 (%0,00), D2 (%0,10), D3 (%0,40), D4 (%1,60) and D5 (%6,40). Materials used in diets; jelatin (G2500-1KG), casein (C3400-1KG SIGMA), dextrin (MP-901520), L-arginine (MP-194626), L-methionine (MP-194707), L-lysine (MP-194696), choline chloride (MP-101386), carboxymethyl cellulose sodium salt (CMC), mineral mixture were supplied from Mp-Bio company, and vitamin mixture was supplied from Mp biomedical company as a mixture from Roche in determined proportions (Table 2).

**Table 2.** Composition of the five experimental diets (g 0.1 kg^−1^)

	Diets				
Ingredients	D1 (Control)	D2	D3	D4	D5
Casein (vitamin-free)	40.00	40.00	40.00	40.00	40.00
Gelatine	8.00	8.00	8.00	8.00	8.00
L-Arginine	0.50	0.50	0.50	0.50	0.50
L-Methionine	0.40	0.40	0.40	0.40	0.40
L-Lysine	0.80	0.80	0.80	0.80	0.80
Fish meal	5.00	5.00	5.00	5.00	5.00
Dextrin	9.10	9.10	9.10	9.10	9.10
Wheat meal	14.98	14.88	14.58	13.38	8.58
Royal jelly	0.00	0.10	0.40	1.60	6.40
Cod liver oil	10.00	10.00	10.00	10.00	10.00
Soybean lecithin	4.00	4.00	4.00	4.00	4.00
Vitamin mix^a^	2.00	2.00	2.00	2.00	2.00
Mineral mix^b^	3.00	3.00	3.00	3.00	3.00
Carboxymethylcellulose	2.00	2.00	2.00	2.00	2.00
Ascorbic acid^c^	0.06	0.06	0.06	0.06	0.06
Choline chloride	0.17	0.17	0.17	0.17	0.17
Proximate composition					
Crude protein	45.68	45.69	45.74	45.92	46.65
Crude lipid	14,25	14,25	14,27	14,34	14.63
Ash	3.84	3.84	3.84	3.84	3.84
Moisture (%)	2.30	2.22	2.41	2.45	2.40

^a^Roche Performance Premix (Hoffman-La Roche, Inc., Nutley, NJ) per gram: vitamin A, 2645.50 IU; vitamin D3, 220.46 IU; vitamin E, 44.09 IU; vitamin B12, 13 mg; riboflavin, 13.23 mg; niacin, 61.73 mg; d-pantothenic acid, 22.05 mg; menadione, 1.32 mg; folic acid, 1.76 mg; pyridoxine, 4.42 mg; thiamine, 7.95 mg; d-biotin, 0.31 mg.

^b^Bernhart Tomarelli salt mixture (ICN Pharmaceuticals, Costa Mesa, CA) g 0.1 kg-1: calcium carbonate, 2.1; calcium phosphate dibasic, 73.5; citric acid, 0.227; cupric citrate, 0.046; ferric citrate (16–17% Fe), 0.558; magnesium oxide, 2.5; manganese citrate, 0.835; potassium iodide, 0.001; potassium phosphate dibasic, 8.1; potassium oxide, 6.8; sodium chloride, 3.06; sodium phosphate, 2.14; zinc citrate, 0.133. Five milligrams of Se in the form of sodium selenite was added per kilogram of the salt mixture.

^c^Phosphitan C (Mg-L-ascorbyl-2-phospahte), Sigma, Germany.

### 2.3. Acclimatisation, expermental design and feeding

A total of 250 fish, 10 (5♀, 5♂) for each of the 25 research tanks, were placed in the tanks. During the experiment, the fish were exposed to the photoperiod with the water temperature at 26 ± 1 °C and for 14 h of light and 10 h of dark. Fish were subjected to an acclimatisation period of two weeks. During this period, fish were fed with basal diet. Zebrafish in five different experimental groups were fed daily up to 1, 2, 5, 8 and 10% of their live weight during the first eight weeks of feeding studies. During the second 8 weeks of feeding, the zebrafish were fed 5% of their live weight gain. The feedings were repeated four times a day at 09:00, 12:00, 15:00 and 18:00.

### 2.4. Tissue sampling

Tissue samples were taken by applying hypothermia to the zebrafish after 56 days of feeding. At the beginning and end of the experiment, three zebrafish in each experiment group were randomly selected in order to measure enzyme activity and gene expression levels. Liver, kidney and muscle samples were then transferred placed in sterile 2 ml eppendorf tubes and stored at −80 °C by adding RNA Later Stabilisation Regent solution (QIAGEN^TM^).

### 2.5. RNA isolation and cDNA synthesis

Total RNA of frozen liver tissues (50 mg) was isolated with a RNeasy Lipid Tissue Mini Kit (Qiagen, cat. no. 74804) using the Qiacube robot (Qiagen, Hilden, Germany). In order to prevent genomic contamination, RNA was treated with DNase I. Quality and concentrations of RNA were verified using spectrophotometer (Nanodrop) and RNA gel electrophoresis, respectively. Subsequently, cDNA was synthesised using the ThermoScript™ RT-PCR System for First-Strand cDNA Synthesis Kit (Invitrogen) in accordance with the manufacturer's protocol. All cDNA was kept at −20 °C until use.

### 2.6. Taqman probe and primer design

For design of primers and TaqMan probes Primer3 software (v. 0.4.0) (http://frodo.wi.mit.edu/) was used with zebrafish SOD (GenBank accession no. FJ807962.1), CAT (GenBank accession no. AF170069.1), GPx (GenBank accession no. AY216589.1) GR (GenBank accession no. NM_001020554) and GST (GenBank accession no. AF285098.1) sequences and BLASTed in order to verify correct mRNA sequences. *β*-Actin (GenBank accession no. AF057040.1) was chosen as an appropriate reference gene due to not being affected by any of the treatments. Real time PCR was performed by conjugating TaqMan probe of the reference gene with Cy5/Blackhole Quencher 2, the fluorophore and quencher molecules. Conjugation of the probes for target genes were performed with FAM/TAMRA. The primer and probe sequences are given in Table 3.

**Table 3.** Sequence, amplification length, genbank accession number of primers and probes used for real-time PCR.

Gene	Primer/Probe	Sequence (5′–3′)	Amplification length (bp)	Accession no
GR	Forward	CTCTCGTCCAGGCGTCTATG	120	NM_001020554
	Reverse	GGGGTGAAAGAAGTGGTGTAAAC		
	Prob	^FAM-^ GCGGACGAGCCCTTCTGACACCT ^-TAMRA^		
CAT	Forward	TTGAGCATGTTGGAAAGACGAC	139	AF170069.1
	Reverse	TGCCCTCATCGGTGTAGAAC		
	Prob	^FAM^- GGCGGGTGAGGCTGGGTCATCA -^TAMRA^		
GST	Forward	TCTCAAGAGCTTCGTGGACAA	115	AF285098.1
	Reverse	AGTGTTGATTTACTGTTTGCCGT		
	Prob	^FAM^- GCCCGTCCCAAAGTCAAAGCTCTGC -^TAMRA^		
GPx	Forward	ACCCAGATGAACGAGCTCCA	89	AY216589.1
	Reverse	TTCTCCTGGTGCCCGAACT		
	Prob	^FAM^- GGGCTGGTGGTTCTGGGCGC -^TAMRA^		
SOD	Forward	CTCCCAGAGGTCAAGCTGTTT	99	FJ807962.1
	Reverse	CTCCTCATCTGCTCCCGTTC		
	Prob	^FAM^- CTGGGGCGTGCGGTGGTGGT -^TAMRA^		
*β*-Actin	Forward	CCTCTCTTGCTCCTTCCACC	150	AF057040.1
	Reverse	TACTCCTGCTTGCTGATCCAC		
	Prob	^CY5^- GGCCTCCCTGTCCACCTTCCAGC-^BQ2^		

### 2.7. Real-time PCR

Gene expression was quantified via real-time PCR analysis by using a real-time PCR cycler Rotor-Gene Q (Qiagen). The real-time PCR was performed in a reaction including template DNA, 900 nM of forward and reverse primers (both target and reference), 250 nM TaqMan probes (both target and reference) and 25 µl FastStartTaqMan Probe Master (Applied Biosystems) which consists of AmpliTaq Gold DNA Polymerase, AmpErase uracil N-glycosylase (UNG), dNTP with dUTP, and optimised buffer component, in a total volume of 50 µl. Thermal cycling conditions for amplification and detection of the samples and the standards were as follows: 50 °C for 2 min for activation of optical AmpErase UNG enzyme, 95 °C for 10 min as hot start to activate AmpliTaq Gold DNA polymerase followed by 45 cycles of denaturation at 95 °C for 15 s, and annealing and extension at 60 °C for 1 min. For evaluation of real-time PCR data, the efficiency (e)(−ΔCt) method^19^ was applied, which is used to determine mRNA levels in gene expression compared to control group and reference gene β-actin. Analytical sensitivity was verified by running standard curves. In order to calculate the amplification efficiency (e), the formula e = 10 (−1/slope) was used based on the slopes of the curves (slope)^19^, and the slope value via Stratagene MxPro3000 software.

### 2.8. Enzyme assays

Following the washing of the samples three times with 50 mM Tris-HCl + 0.1 M Na_2_SO_4_ (pH 8.0), each sample was homogenised in liquid nitrogen. Samples then transferred to the same buffer, and centrifuged at 15,000 g for 60 min at 4 °C. Further studies were performed with the supernatant. Below mentioned enzyme activities were spectrophotomethrically measured.

#### 2.8.1. SOD activity

Superoxide dismutase (SOD, EC 1.15.1.1) activity was analysed by inhibition of xanthine/xanthine oxidase generated O_2_^−^ reduction of nitrobluetetrazolium (NBT)^20^. O_2_^−^ reduced the NBT to blue formazan which shows a strong absorbance at 560 nm. One unit was defined as the amount of protein which generated the 50% percent inhibition of reaction. 1 ml of reaction mixture included 1 M Tris-HCl, 5 mM EDTA buffer (pH 8.0), 150 µM NBT, 400 mM Na_2_CO_3_, 1 g L^−1^ bovine serum albumine, 3 mM L^−1^Xanthine, 0.833 U mL^−1^Xanthine oxidase, 0.8 mM CuCl_2_ and tissue homogenate (30 µL).

#### 2.8.2. GPx activity

Glutathione peroxidase (GPx, EC 1.11.1.9) activity was assayed by monitoring the oxidation rate of nicotinamide adenine dinucleotide phosphate (NADPH) at 340 nm by the coupled reaction with glutathione reductase (GR)^21^^,^^22^. The reaction tube contained 1 M Tris-HCl, 5 mM EDTA buffer (pH 8.0), 0.1 M GSH, 10 U mL^−1^ GR, 2 mM NADPH, 7 mM t-butylhydroperoxide, distillated water and tissue homogenate (10 µL).

#### 2.8.3. GST activty

Glutathione *S-*transferase (GST, EC 1.5.1.18) activity was assayed with a reaction mixture containing 1-chloro-2,4-dinitrobenzene (CDNB) as substrate, 0.11 M phosphate buffer (pH 6.5), 30 mM CDNB, 0.1 M GSH, distillated water and tissue homogenate (50 µL)^23^. The change in optical density at 340 nm was measured as the activity rate (ε = 9.6 mM^−1 ^cm^−1^).

#### 2.8.4. CAT activity

Catalase activity was measured by modifying the method by Aebi^24^. The homogenate preparation procedure used for the SOD enzyme has been used for the specific catalase enzyme activity measurement. The absorbances of the homogenates were measured at 240 nm for 1 min using 3 ml quartz cuvettes.

#### 2.8.5. GR activity

GR activity was assayed spectrophotometrically at 340 nm using the method reported by Carlberg and Mannervik^25^. Then, specific activity (EU/mg) was calculated by combining these values with protein determination results.

### 2.9. Protein determination

Bradford's method^26^ was used to determine the quantitative proten amount spectrophotometrically at 595 nm, with bovine serum albumin as a standard.

### 2.10. Statistical analysis

Results were given as mean ± SD (*n* = 5). Each experimental unit (aquarium) was regarded as a replicate. Two fish from each aquarium were used for analysis of enzyme activity. In order to test the effect of royal jelly on enzyme activity and expression level, data were evaluated with one way analysis of variance (ANOVA) and means were subsequently compared by Duncan’s multiple range tests. Activity and expression level of each enzyme was compared with independent *t* test for each experimental group with those for initial fish. Differences at *P* < 0.05 were considered significant.

## 3. Results

**Figure 2.**
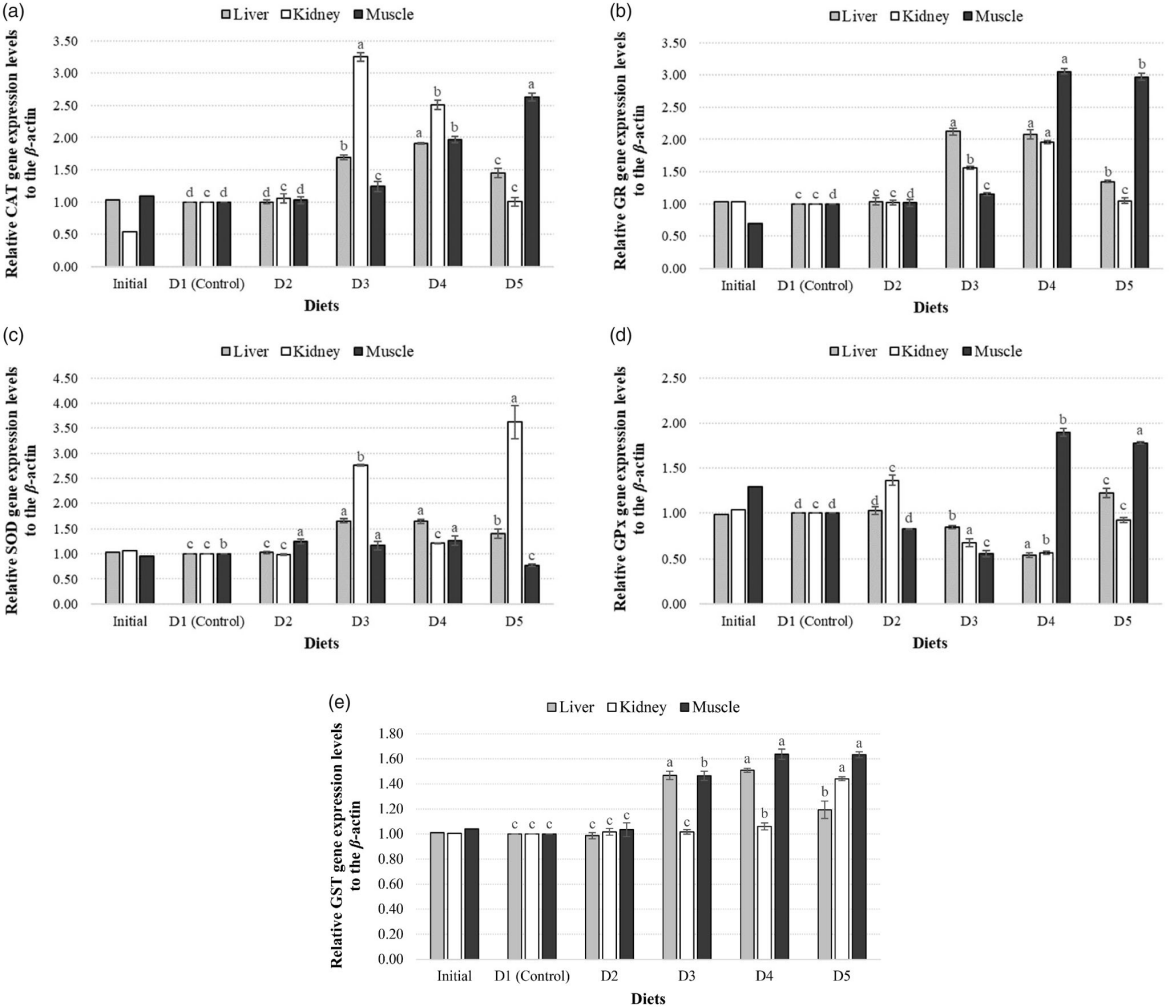
Expression levels of CAT (a), GR (b), SOD (c), GPx (d) and GST (e) genes in three different tissues of zebrafish among experimental diet groups.

**Figure 3.**
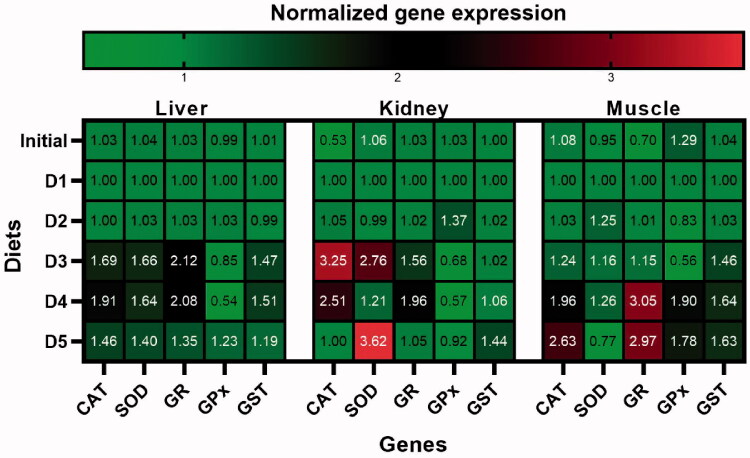
Gene expression heat map following different diet exposures. Values depict mean fold change (RQ) values versus control group. The bar is the colour key depicting fold change levels. Green signifies down-regulation and red upregulation. (For interpretation of the references to colour in this figure legend, the reader is referred to the Web version of this article.).

**Figure 4.**
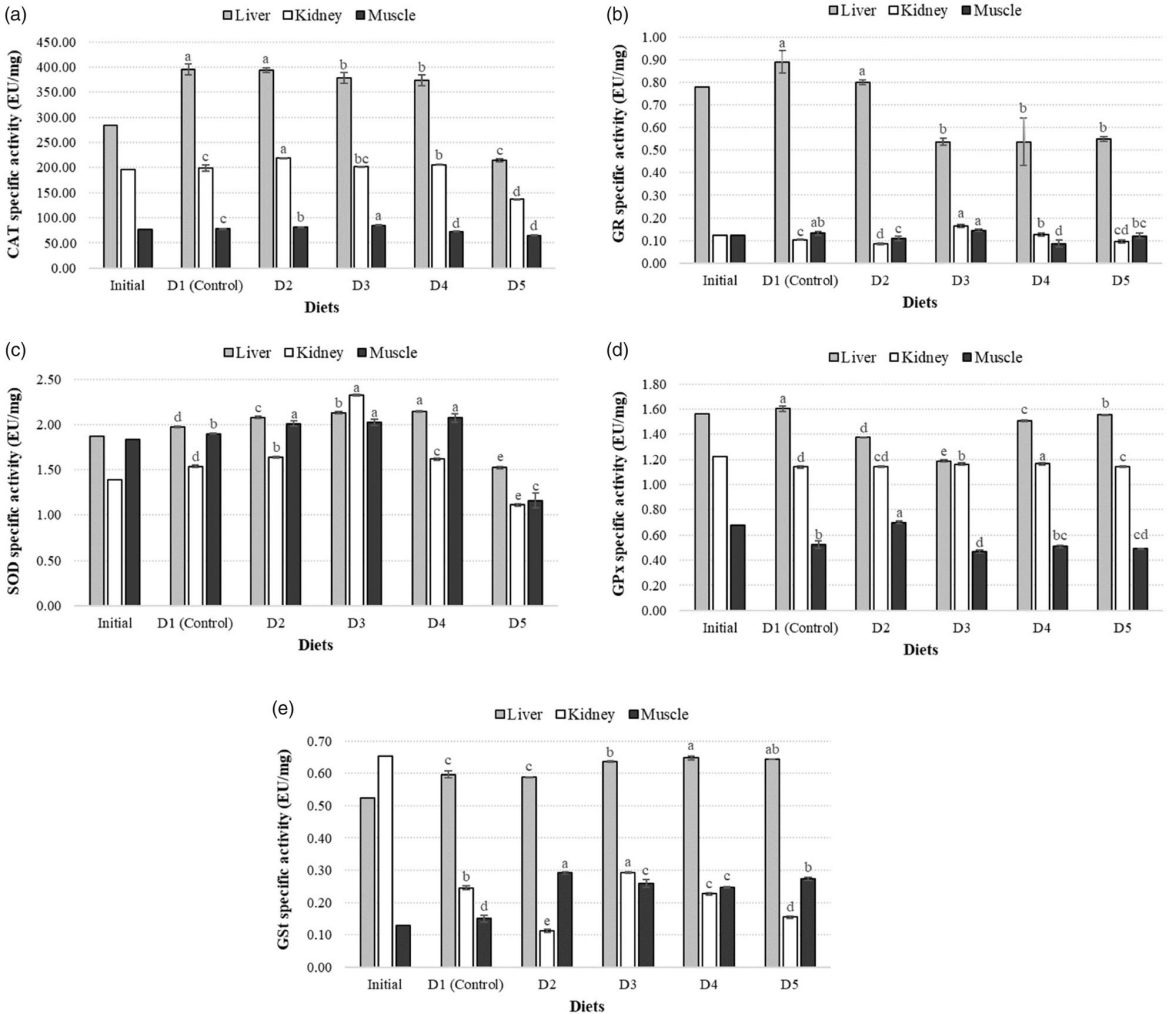
Specific activity of CAT (a), GR (b), SOD (c), GPx (d) and GST (e) genes in three different tissues of zebrafish among experimental diet groups.

Dietary treatments had significant effects on gene expression and activity of antioxidant enzymes (Figures 2– 4).

### 3.1. Gene expression results

#### 3.1.1. CAT mRNA expression levels in muscle, liver and kidney tissues

CAT expression levels in D3, D4 and D5 groups were found to be significantly increased compared to the control group in muscle tissue. However, difference between D2 group and control was not statistically significant. In liver, CAT expression levels increased in D3, D4 and D5 groups compared to the control and the highest elevation was found in D4. The change in D2 group was not significant compared to the control. In kidney, expression levels of D3 and D4 groups were determined to increase compared to the control group, while the change in D2 and D5 groups was not significant (Figure 2).

#### 3.1.2. SOD mRNA expression levels in muscle, liver and kidney tissues

SOD gene expression levels of muscle tissue were found to increase in D2, D3 and D4 groups compared to control with the highest increase in D4 group. However, mRNA level of D5 group was found to decrease compared to the control. In liver, while D2 group did not show a significant variation, mRNA levels of D3, D4 and D5 groups exhibited a significant increase, and the highest mRNA level was found in D3. In kidney, the changes in SOD expression levels of D2 and D4 diet groups were not significant compared to the control group while it was found to increase significantly in D3 and D5 groups (Figure 2).

#### 3.1.3. GR mRNA expression levels in muscle, liver and kidney tissues

Gene expression levels in muscle tissue were determined to be increased in D3, D4 and D5 groups compared to control, and the highest increase in mRNA level was in D4 group. However, D2 group did not show a significant change compared to control. In liver, GR mRNA levels increased significantly in D3, D4 and D5 groups compared to control, while D2 did not show a significant difference. In kidney, an increase in D3 and D4 diet groups was detected compared to control, while D2 and D5 groups did not show a significant change (Figure 2).

#### 3.1.4. GPx mRNA expression levels in muscle, liver and kidney tissues

GPx gene expression levels in muscle tissue were determined to decrease in D2 and D3 groups compared to control, and to increase in D4 and D5 groups. The maximum increase was seen in D4 group, while D3 diet group exhibited the lowest expression. In liver, mRNA levels of D3 and D4 groups were found to significantly decrease compared to the control group, while an increase was seen in D5 group, but no significant difference in D2 group. In kidney, the highest expression was detected in D2 group. However, mRNA levels of D3, D4 and D5 groups decreased compared to control, and minimum level was found in D4 group (Figure 2).

#### 3.1.5. GST mRNAexpression levels in muscle, liver and kidney tissues

In muscle, GST expression levels of D3, D4 and D5 groups increased significantly compared to control, and the highest mRNA level was shown to be in D4. However, D2 group did not show a significant change. In liver, GST mRNA levels of D3, D4 and D5 groups were found to increase compared to the control, and D4 group showed the highest level. Expression level of D2 group was lower than other groups with no significant difference from the control group. In kidney, the difference between the control group and D3 group was not found to be significant. However, the increase of mRNA level in D4 group was significant and the increase in D5 group was more than all diet groups (Figure 2).

#### 3.1.6. Hierarchical clustering of gene expression levels

Hierarchical clusters of expression profiles of antioxidant enzyme genes (SOD, CAT, GPx, GR, GST) in all diet groups are shown in Figure 2. It can be concluded that, GPx-GR mRNA levels are similar in muscle tissue, SOD-GST in liver tissue and GPx-GST in kidney tissue (Figure 3).

### 3.2. Enzyme activities

#### 3.2.1. CAT activity in muscle, liver and kidney tissues

In muscle, CAT activity of D2 and D3 diet groups were found to increase significantly compared to the control while activities in D4 and D5 groups were decreased. In liver, the highest CAT activity was determined in control group with an insignificant difference compared to D2 group. Enzyme activites of D3, D4 and D5 groups decreased compared to control and the lowest activity was in D5 group. In kidney, enzyme activities of D2, D3 and D4 groups significantly increased compared to control group. The lowest enzyme activity was found in D5 group with a statistically significant decrease from the control (Figure 4).

#### 3.2.2. SOD activity in muscle, liver and kidney tissues

SOD activity of D2, D3 and D4 groups in muscle tissue increased significantly compared to control, while a decrease was detected in D5 group. In liver, D2, D3 and D4 groups showed a statistically significant increase compared to the control group, while activity of D5 decreased. The highest activity among all groups was found in D4 group. SOD activites of D2, D3 and D4 groups in kidney tissue showed a significant increase compared to control, while a decrease was found in D5 group. The highest activity among all groups was determined in D3 group (Figure 4).

#### 3.2.3. GR activity in muscle, liver and kidney tissues

The highest GR enzyme activity in muscle tissue was found in D3 diet group with a significant increase. The lowest activity compared to control was in D4 diet group. In liver, D3, D4 and D5 groups showed a significant decrease compared to control, while the decrease in D2 group was not found to be statistically significant. Enzyme activities in all diet groups were decreased compared to control. In kidney, a decrease was detected in D2 group compared to control, while D3 and D4 groups showed significant increase. The activity difference of D5 group was not statistically significant (Figure 4).

#### 3.2.4. GPx activity in muscle, liver and kidney tissues

In muscle tissue, GPx activity of D2 group increased compared to the control, which also have the highest enzyme activity among all groups compared to each other. This increase was statistically significant, while the increase in D4 group was not. D3 and D5 groups showed a significant decrease, and the lowest activity was found in D3. In liver, enzyme activities of all diet groups showed a significant decrease compared to control, and lowest activity was found in D3 group. In kidney tissue, D3, D4 and D5 groups showed a significant increase while D2 did not have a significant difference in GPx activity. The highest activity was detected in D4 group (Figure 4).

#### 3.2.5. GST activity in muscle, liver and kidney tissues

In muscle tissue, GST activity of all groups increased compared to control group and the highest value was found in D2 group. In liver, activities of D3, D4 and D5 groups were found to increase compared to control, and D4 group showed the highest GST enzyme activity. The difference in D2 group was not significant. GST activity of D3 group in kidney tissue increased compared to control, while decrease was detected in D2, D4 and D5 groups (Figure 4).

### 3.3. Correlation between enzyme activity and gene expression levels

**Figure 5.**
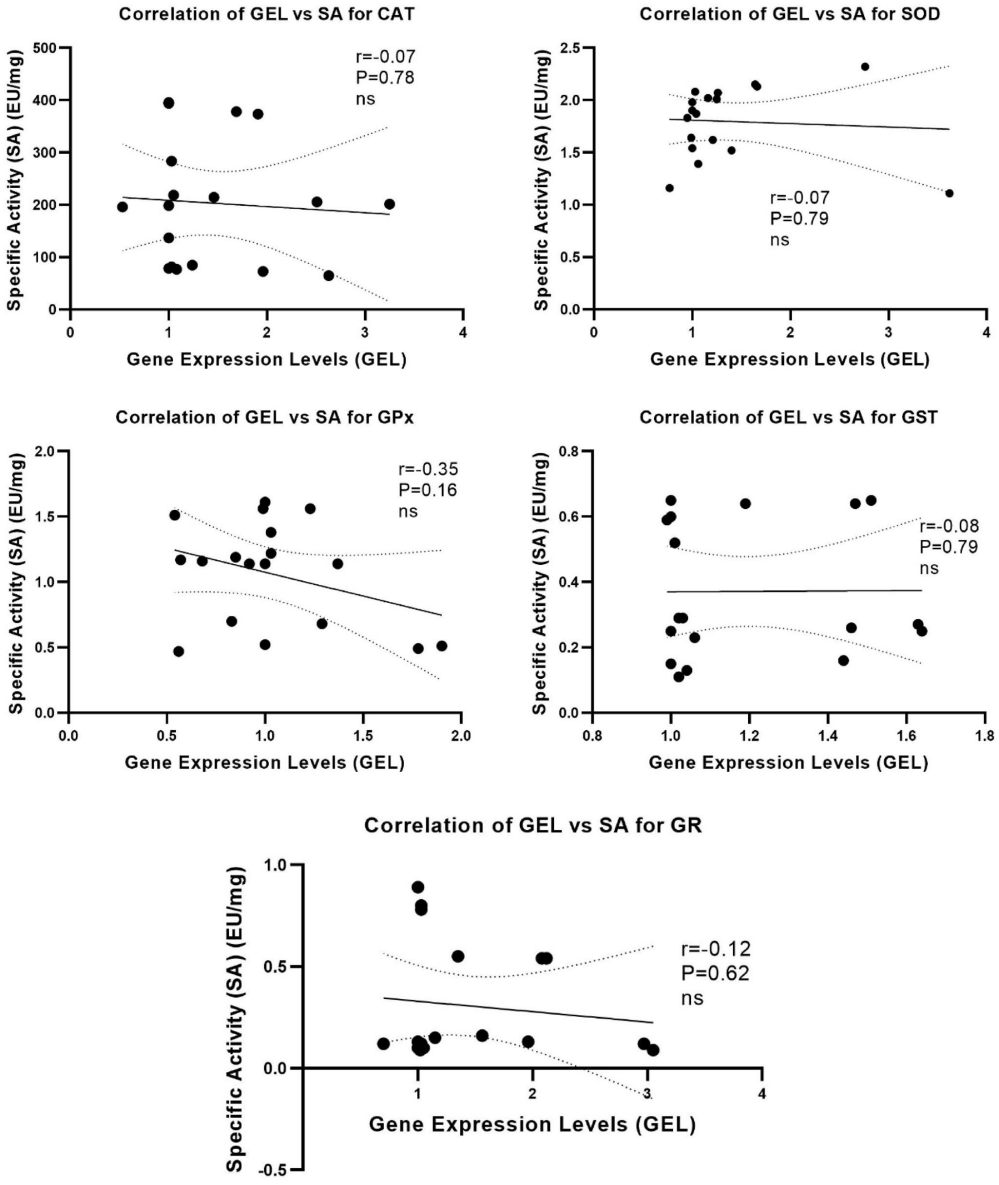
Pearson’s correlation was calculated between the enzyme activity and gene expression levels for CAT, SOD, GPx, GST and GR in all tissues. Any significant correlation was not observed for all enzymes.

Pearson’s correlation was calculated between enzyme activities and gene expression levels for CAT, SOD, GR, GPx and GST in muscle, liver and kidney tissues. As can be seen from the graphs, no significant correlation was observed for activity and mRNA levels of the enzymes (Figure 5).

## 4. Discussion

Bee products such as honey, bee pollen, royal jelly, propolis and bee venom have been focussed by researchers in recent years due to their nutritional properties and positive effects on health^27^^,^^28^.

According to many scientific researches, royal jelly has been reported to stimulate cell proliferation, have antioxidant effects *in vitro* studies, delay ageing and strengthen the immune system. Also, it is widely used as a commercial medical product. There exist limited investigations on royal jelly. In a study conducted by Kanbur et al.^29^, it was determined that administration of fluoridine caused oxidative stress in rats and decreased SOD, CAT and GPx activities. However, administration of royal jelly with fluoridine was found to improve SOD, CAT and GPx activities.

In another study, it has been reported that cisplatin administration to rats caused a decrease in SOD, CAT and GPx activities, but the addition of royal jelly provided an improvement in the activity of the enzymes^30^.

In another study conducted on rats, it was observed that paracetamol administration caused a decrease in GPx activity in rat liver, but the addition of royal jelly increased this enzyme activity^31^.

In a study investigating the antioxidative effect of the royal jelly on *Saccharomyces cerevisiae* yeast cells, the yeast used was enriched with royal jelly at two different ratios (1,2 and 5,0 g/L) and developed in YEPD medium. At the end of the study, it was observed that royal jelly reduced intracellular oxidation in a dose-dependent manner and affected the metabolic activity related to the growth. It was also reported that royal jelly not only acts as a scavenger of reactive oxygen species but also affects protein expressions^9^.

In a study in which different proportions of the royal jelly were added to sheep sperm, were investigated the effects of royal jelly on the kinematics, plasma membrane, NO and antioxidant capacity of sperm samples taken at different time intervals (i.e., 0, 24, 48, 72, 96 and 120 h). Due to the antioxidant properties of the royal jelly, it was reported to have positive effects on the activity, mortality and plasma membrane functions of sheep sperm^32^.

In another study conducted on rats, effectiveness of royal jelly on oxidative stress caused by fumonisins was investigated. Different diets, containing only fumonisin (FB), only royal jelly (RJ), and fumonisin and royal jelly (FB + RJ), were administered to 60 Sprague-Dawley male rats. SOD and GPx activities were significantly decreased in the FB administration compared to control. The activities of these enzymes were increased in the RJ diet compared to control. In the FB + RJ administration, the enzyme activities improved and approached to the control. It was determined that fumonisin caused a decrease in SOD and GPx enzyme activities and the royal jelly compensated this decrease, and it was concluded that oxidative stress caused by fumonisin was also eliminated^33^.

Guo et al.^34^ reported that royal jelly proteins hydrolysed with Protease N showed strong antioxidant activation against linoleic acid peroxidation. Shorter et al.^35^ investigated the effects of increasing concentrations of royal jelly on the viability, development time, body size, productivity, life cycle and large-scale genome transcription amounts of *Drosophila melanogaster*. They reported that low concentrations of the royal jelly made significant differences in body size in both genders, while high concentrations decreased body size, increased mortality, shortened life cycle and decreased productivity. In addition, they found that royal jelly increased the development periods for both genders with increasing concentrations^35^.

Zebrafish is a very suitable model organism for toxicological and nutritional studies, and oxidative stress investigations performed with this organism have gained great interest in the recent years. For instance, in a study to determine the effects of genetically modified plant sources, effects of genetically modified (GM) and non-GM maize diets on feed utilisation, growth and stress were investigated in zebrafish. When both maize sources were compared, it was observed that the growth rate of fish fed GM was higher, but SOD and HSP-70 mRNA expression levels were lower. They reported that effects of GM products differ depending on the gender, and statistically different levels of SOD mRNA were observed in male and female fish^36^.

In a study, 4–6 weeks old zebrafish were used as model organism, and the role of Vitamin E (α-tocopherol) in foetal development was investigated. Fish were fed up to 1 year old with a control diet, α-tocopherol added diet (E+), non-added diet (E-). All groups showed similar growth rates. Vitamin E deficiency was determined to cause neurological damage in adult zebrafish. No variations were found in the expression of oxidative stress and apoptosis genes in their liver for all three diets. High rate of deaths at the 24th hour after fertilisation and anomalies and deaths at the 120th hour were detected in the embryos of the non-vitamin E (E-) group^37^.

In another study on zebrafish; growth, survival rate and condition factors of fish fed for 9 weeks with five different commercial diets and two different diets (prepared in the laboratory) were investigated. Live weight gain in fish-fed laboratory diet was found higher than others. While there was no statistically significant difference between the groups in terms of condition factor, the survival rate was found to be the highest (with 100%) in the group fed commercial diet^38^.

Since studies on the development of zebrafish diets may yield different results in feeding studies, it is recommended to develop novel diets. Furthermore, there is no study investigating the effect of the royal jelly on zebrafish diets. For this reason, our study is the first research on this subject.

Our current study has interesting results. Expression levels of the antioxidant enzymes mostly elevated in royal jelly diets which in turn resulted in a transcriptional regulation. Activity of the enzymes also increased in some of the royal jelly groups compared to the control diet. However, no remarkable correlation was observed between expression levels and activities.

In the muscle tissue, GST activity increased with supplementation of royal jelly in diets. The increase in GST activity caused a decline in GSH levels which might have resulted in downregulated activity of GPx, another GSH dependent enzyme. Moreover, downregulation of GPx activity might have caused deceleration of GSH-GSSG conversion which finally resulted in lower GR activities. The increase of the SOD activity in D2 and D3 groups is in parallel with CAT activity in the same diets. This might result from the elevation of H_2_O_2_ levels produced by SOD reaction.

In the kidney tissue, SOD activity significantly increased in royal jelly groups except for D5, which had the minimum activity even lower than control. Similar results were observed for CAT and GPx which indicates that H_2_O_2,_ produced by SOD, was metabolised successfully. GR activity also increased as expected because of the increasing GSSG concentrations produced by GPx and GST.

In the liver tissue, activity of the enzymes mostly decreased in royal jelly diet groups except for increasing GST activity. This is also an interesting result indicating that detoxification by glutathione conjugation was stimulated in the liver after royal jelly supplementation and probably the detoxifitation products caused inhibition of other enzymes on active protein conformation. Moreover, in all tissues, enzyme activities mostly decreased in D5 group which has the highest royal jelly concentration. This might have resulted from feedback inhibition of the products. Overall results show that royal jelly alters both expression and activity of the antioxidant enzymes in dose and tissue-dependent manner.

There exist different fish feeding studies in the literature, but no studies have been done about royal jelly as a fish nutrient. Although there are many investigations on the effects of royall jelly on antioxidant enzyme activities, very limited research has been performed on both expression and activity of the enzymes. In fact, it is important for toxicologist and pharmacologists to see the influences on both mRNA levels and protein activity. Thus, our findings could make significant contributions for researchers interested in oxidative stress and feeding.

## 5. Conclusion

Several diets have been discovered in order to provide specific nutritional needs of different fish species. Although antioxidant features of royal jelly have been well established, diets including this antioxidant have been rarely analysed in fish. Our study aimed to constitute a relationship between royal jelly administration and oxidative stress enzymes in zebrafish both at transcriptional and activity levels. We have demonstrated that inclusion of royal jelly in zebrafish diets modulated both mRNA expression levels and activity of the enzymes in dose and tissue-dependent manner without significant correlations between gene expression and activities.

It is crucial to improve feed quality of fish species in order to sustain the future of aquaculture because of the increasing demand. Therefore, it is important to perform more investigations on fish diets including natural products in order to obtain healthy, protective, easy access and more beneficial nutrients. Royal jelly represents a promising class of fish nutrient and results of our study may assist scientists to design novel fish diets with enhanced efficiency and other tailored properties.
